# Prosocial behaviours and emotional intelligence as factors associated with healthy lifestyles and violence in adolescents

**DOI:** 10.1186/s40359-024-01559-2

**Published:** 2024-02-22

**Authors:** Alba González Moreno, María del Mar Molero Jurado

**Affiliations:** Department of Psychology, Developmental and Educational Psychology Area, University of Almería, Ctra. La Cañada de San Urbano s/n, C.P. 04120 Almería, Spain

**Keywords:** Adolescence, Emotional intelligence, Prosocial behaviour, School violence, Substance use

## Abstract

Adolescence is a stage of life characterised by vulnerability, which shapes young people’s trajectories and potentially influences their behaviour. In this crucial period, the promotion of prosocial behaviours and the development of emotional intelligence are understood as key factors influencing adolescents’ psychological and personal well-being. The general objective of this study was to find out the relationship between these two variables - prosocial behaviours and emotional intelligence - and their correlation with the maintenance of a healthy lifestyle and another fundamental aspect such as violence among young people in the academic context. A total of 743 secondary school students participated in this research with a descriptive-cross-sectional design. This study used several instruments, including an ad hoc questionnaire to assess socio-demographic aspects and school violence, the Prosocial Behaviour Questionnaire (PBQ), the TMMS-24 for the assessment of Emotional Intelligence, the Healthy Lifestyles Questionnaire (HLQ-II) and the Satisfaction with Life Scale (SLS). The results highlighted a convincing link between the display of prosocial behaviours and emotional intelligence with various dimensions of healthy living such as healthy diet or respect for mealtimes. In particular, the research revealed a pronounced correlation between adolescents who showed greater emotional repair and respect and their lower involvement in school violence and substance use. In addition, the likelihood of having a healthy life was found to be linked to variables such as being male, respect, social relationships and emotional repair. In contrast, the likelihood of having an excessive consumption of certain harmful substances such as alcohol or tobacco was found to be linked to age, empathy and emotional clarity. These results highlight the crucial role that prosocial behaviours and emotional intelligence play in shaping adolescents’ lives. In conclusion, the need to promote such variables as prosocial behaviours and emotional intelligence in adolescent students in order to promote healthy lifestyles and reduce school violence and substance use in this age group is discussed.

## Introduction

Adolescence is a developmental stage characterised by various physical, psychological and social changes [1]. This series of changes can produce certain externalised problems in young people, which have a negative impact on their development, mental health and social, personal and school adjustment [2]. This stage of adolescence is a period of susceptibility and vulnerability, and young people tend to be socially influenced to engage in both prosocial and antisocial behaviour [3]. Aspects such as empathy or difficulty in stress management act as risk factors for the use of psychoactive substances such as tobacco, alcohol and cannabis [4]. On the other hand, it is noted that personal well-being promotes positive behaviours and self-esteem in adolescents [5]. Therefore, it is necessary for adolescents to have the necessary tools and skills to perform adequately [6].

### Prosocial behaviours in adolescence

Prosocial behaviours refer to voluntary attitudes that are performed with the aim of establishing positive interpersonal relationships and sustaining social and personal well-being [7]. Research indicates that when young people engage in prosocial acts with their peers, they experience a more positive mood, which is even more beneficial for those suffering from depression [8]. The same is true for the influence of engaging in sporting activities, as they are estimated to promote prosocial values, increased psychological well-being, moral development, social skills and reduced levels of stress and aggression in students [9]. This relationship between healthy lifestyle and prosocial behaviours is positively associated with aspects such as healthy eating and negatively related to unhealthy foods, depression, stress and anxiety [10]. Such a negative relationship is also shown in the association between prosocial behaviours and substance use, as it is estimated that prosocial adolescents tend to have lower alcohol and/or tobacco use [11].

It has been shown how prosocial behaviour in adolescents acts as a mediating variable in relation to peer victimisation and aggressive behaviour [12]. This assertion is reflected in the study provided by Cardozo [13], as it indicates that adolescent bullies have a negative correlation with prosocial behaviours; while they do have a positive significance with antisocial behaviours. The study also shows that young people who are not involved in school violence and those who are victims have high scores on prosocial behaviour such as empathy, respect and leadership, and low scores on delinquent behaviour. In line with these results, prosocial behaviour is significantly and negatively associated with violence [14].

### Emotional intelligence and its impact on adolescence

Referring to emotional intelligence, this construct is understood as the mental capacity to understand, perceive, use and regulate one’s own and others’ emotions [15]. Current studies indicate that people with good levels of emotional intelligence have better psychological adjustment such as higher self-esteem and happiness and less depression [16, 17], as well as being more satisfied with life [18]. Emotional intelligence is also shown to be positively associated with physical activity and negatively associated with anxiety levels [19]. About the school context, it is noted that emotional intelligence promotes interpersonal relationships and can therefore be considered a protective factor against school violence [20]. Furthermore, it is estimated that emotional intelligence has a negative relationship with different types of aggression [21], as well as emotions help to reduce violent behaviour in adolescents [22]. The same occurs with the consumption of psychoactive substances, as it is noted that emotional intelligence acts as a mediator in the consumption of substances such as alcohol [23].

### Aim and hypothesis of the study

Current scientific evidence has not evaluated the impact of prosocial behaviours and emotional intelligence in adolescence together. Thus, this research aims to assess whether both constructs promote a healthy lifestyle and life satisfaction in adolescence, as well as whether they act as moderators of school violence and substance use. The initial hypotheses considered are the following:

#### H1

Prosocial behaviours and emotional intelligence are positively associated with healthy lifestyle habits and life satisfaction.

#### H2

There are differences according to prosocial behaviour and emotional intelligence in the roles of school violence and in alcohol and tobacco consumption.

#### H3

The healthy living group and the non-consumption group are linked to prosocial behaviours and emotional intelligence.

#### H4

Prosocial behaviours and emotional intelligence are associated with an increased likelihood of healthy living and reduced substance use.

## Method

### Study design and participants

This quantitative research was carried out using a descriptive cross-sectional design. To verify the reliability of the study, the guidelines provided by the STROBE statement for cross-sectional studies were followed [24]. The study sample consisted of a total of 743 young people aged 14–19 years (*M =* 14.99; *SD =* 0.86), 50.7% of whom were female (*n =* 377) and 49.3% male (*n =* 366). All participants were secondary school students from different secondary schools in the province of Almería (Spain), 50.7% of whom were in their third academic year and 49.1% in their fourth year. Most of the adolescents were of Spanish nationality (92.9%), although students of other nationalities such as Moroccan, Argentinian or Russian also participated. This sample was randomly selected by identifying different high schools in the province of Almería. Subsequently, students were randomly selected from each school to reach the desired sample size. Inclusion criteria were being between 14 and 19 years old and enrolled in the third or fourth year of secondary education. Exclusion criteria included refusal to participate, lack of informed consent and the presence of conditions that could affect understanding or active participation in the study.

### Instruments

The measurement of the different variables in adolescents was carried out using a booklet containing a series of ad hoc questions and validated instruments. The variables in this study were assessed as follows:

#### Prosocial Behaviour

Adolescents’ prosocial behaviour has been assessed using the original Spanish version of the Prosocial Behaviour Questionnaire (PCQ) [25]. This questionnaire is aimed at adolescents aged 10–17 years and examines participants’ use of certain helping behaviours such as sharing, collaborating, understanding and encouraging. This instrument has a total of 55 items consisting of four dimensions and is answered on a Likert scale with four response options from “never” to “always”. The first dimension is Empathy, which has a total of 20 items and refers to a person’s ability to put him/herself in the place of another person and alleviate his/her discomfort (e.g. “I help those who have problems”). The second dimension, Respect, consists of 16 items and refers to the ability to treat others in the right way (e.g. “When I make a mistake, I know how to recognise it”). The third dimension, called Social Relationships, has 11 items and focuses on the ability to form positive social connections (e.g., “I like to talk to my friends and colleagues”). Finally, the fourth dimension is Leadership and refers to the ability to lead and organise team activities (e.g., “When something needs to be done, I take the initiative to start”). This inventory does not establish an overall score but establishes individual scores for each of the dimensions. The internal consistency of these dimensions was acceptable and excellent: Empathy (*α =* 0.90), Respect (*α =* 0.78), Social relations (*α =* 0.69) and Leadership (*α =* 0.74).

#### Emotional Intelligence

This variable was analysed using the *Trait Meta-Mood Scale* (TMMS-24) validated in Spanish [26] from its original scale [27]. This scale has a total of 24 items classified into three dimensions and is answered on a five-point Likert scale (1 = Not at all agree; 2 = Somewhat agree; 3 = Fairly agree; 4 = Strongly agree; 5 = Strongly agree). The reliability obtained by means of this questionnaire was good in each of its dimensions: Factor 1.- Attention (*α =* 0.89); e.g. “I pay a lot of attention to feelings”, Factor 2.- Clarity (*α =* 0.88); e.g. “I usually know my feelings about people”, Factor 3.- Repair (*α =* 0.85); e.g. “I try to have positive thoughts even if I feel bad”.

#### Healthy life

This construct was assessed by means of the Healthy Lifestyles Questionnaire (CEVS-II) validated in the Spanish population [28]. This questionnaire contains 27 items grouped into seven dimensions: Healthy diet (e.g., “I consider that I have a balanced and healthy diet”); Respect of mealtimes (e.g., “I usually respect the schedule of the main meals of the day”); Rest habits (e.g., “I sleep enough hours so that my body is rested”); Tobacco consumption (e.g., “I feel good when I smoke”); Alcohol use (e.g. “Alcohol makes me have a better time”); Use of other drugs (e.g. “I like the way I feel when I take drugs”) and Physical activity (e.g. “I consider myself a physically active person”). The entire instrument is answered on a five-point Likert scale ranging from 1 (strongly disagree) to 5 (strongly agree). The internal consistency of the scale was good on each of its dimensions: Healthy diet (*α =* 0.59), Respecting mealtimes (*α =* 0.65), Rest habits (*α =* 0.78), Tobacco use (*α =* 0.89), Alcohol use (*α =* 0.86), Use of other drugs (*α =* 0.81) and Physical activity (*α =* 0.82).

#### Satisfaction with life

This variable has been measured using the Spanish adaptation of the Satisfaction with Life Scale [29] originally created by Diener [30]. This instrument is one of the most widely used to measure people’s quality of life using their own criteria [31]. The unidimensional scale is composed of five items (e.g. “In most respects my life is the way I want it to be”) which are answered on a five-point Likert scale (1 = strongly disagree and 5 = strongly agree). The total of the responses obtained is ranked between 5, understood as very dissatisfied with your life, and 25, which would indicate very satisfied with your life. In this study, the internal consistency of this scale was good (*α =* 0.84).

#### School Violence

Students’ perception of school violence has been examined by means of questions incorporated in the ad hoc. These questions are answered dichotomously and are focused on three roles: victim (e.g. “Have you suffered episodes of violence from your classmates?“), bully (e.g. “Have you ever been violent towards your classmates?“) and observer (e.g. “Have you observed situations of violence towards other classmates?“).

#### Alcohol and/or tobacco use

The use of these substances was examined by means of ad hoc questions to find out whether and how often participants use these substances.

### Procedure and data collection

Once the instruments had been selected and the data collection booklet had been prepared, we contacted several secondary schools in different municipalities in the province of Almería (Spain). A total of six schools agreed to participate in the study, so an appointment was made with the school management to attend one day and have the students fill in the booklets themselves. Before data collection began, all students and their legal guardians were informed of the purpose of the research and gave their consent to participate in the research. This collection took place from February to June 2022. This study has been approved by the Bioethics Committee on Human Research of the University of Almeria with reference UALBIO2021/025.

### Data analysis

Data analysis was carried out using SPSS statistical software version 28 [32]. Cronbach’s alpha coefficient was used to test the reliability of the instruments used. This coefficient is interpreted as follows: <0.5 unacceptable, > 0.5 poor, > 0.6 questionable, > 0.7 acceptable, > 0.8 good and > 0.9 excellent [33].

A descriptive analysis was conducted to provide relevant information about the students who participated in this study such as age, gender or nationality. In addition, a Pearson’s bivariate correlation analysis was performed to determine whether there was a relationship between the variables studied. This analysis was used to determine the correlations between the main variables of the study (prosocial behaviours and emotional intelligence) and the variables to be studied: healthy living and life satisfaction. The absolute values obtained are interpreted according to the following categories: no correlation between 0 and 0.10; weak correlation between 0.10 and 0.29; moderate correlation between 0.30 and 0.50; and finally, strong correlation between 0.50 and 1.00 [34].

In addition, Student’s t-test for independent samples was performed. This test was performed to examine differences according to the role of violence and substance use (tobacco or alcohol) by adolescents on the variables of prosocial behaviours and emotional intelligence. Cohen’s was also calculated to estimate the effect sizes: small 0.50; medium 0.50–0.80; and large ≥ 0.80 [35].

To identify different profiles according to the scores obtained in the dimensions of the healthy lifestyle habits questionnaire, two-stage cluster analysis was applied (a first analysis for the dimensions related to healthy habits and another for substance use habits). Once the groups or clusters were identified, a comparative analysis of means was carried out using Student’s *t-test* for independent samples and Cohen’s *d* statistic to check the effect size of the differences found. Through this test it has been possible to establish four differentiated groups (healthy adolescents / unhealthy adolescents; adolescent consumers / adolescent non-consumers) and to find out the differences between these groups in prosocial behaviour and social skills.

Finally, a binary logistic regression analysis was carried out to determine the probability of having a healthy lifestyle and a habit of consuming harmful substances such as tobacco, alcohol or other drugs according to the variables of sex, age, prosocial behaviour and emotional intelligence. Therefore, by means of this test we were able to find out which variables are more likely to promote a healthy lifestyle and a lower consumption. Statistical significance was set at a *p-value of* less than 0.05.

## Results

### Descriptive analyses and correlations

The results obtained about the correlations between the variables examined are shown in Table 1. These results indicate how all the dimensions of prosocial behaviour (empathy, respect, social relations and leadership) are significantly related to the dimensions of emotional intelligence (attention, clarity and repair) and to life satisfaction. On the other hand, in terms of the relationships between prosocial behaviour and healthy lifestyle, it was found that the empathy dimension correlates with healthy diet, respect for mealtimes and rest habits; the respect dimension correlates positively with healthy diet, respect for mealtimes, rest habits and physical activity, and negatively with smoking, alcohol and other drug use; finally, both the social relations and leadership dimensions correlate positively with healthy diet, respect for mealtimes, rest habits and physical activity.

**Table 1:** Descriptives and correlation matrix between prosocial behaviour, emotional intelligence, healthy lifestyle and life satisfaction (*N =* 743)

		[1]	[2]	[3]	[4]	[5]	[6]	[7]	[8]	[9]	[10]	[11]	[12]	[13]	[14]	[15]
Prosocial Behaviour (PCC)	[1] Empathy	-														
	[2] Respect	0.53***	-													
	[3]Social Relations	0.39***	0.28***	-												
	[4]Leadership	0.35***	0.16***	0.53***	-											
Emotional Intelligence (TMMS-24)	[5]Attention	0.38***	0.23***	0.98**	0.15***	-										
	[6]Clarity	0.20***	0.27***	0.36***	0.41***	0.29***	-									
	[7]Repair	0.28***	0.35***	0.41***	0.45***	0.23***	0.53***	-								
Healthy Lifestyle (CEVS-II)	[8]Healthy Diet	0.08*	0.19***	0.20***	0.17***	0.02	0.19***	0.24***	-							
	[9]Respecting Mealtimes	0.08*	0.22***	0.19***	0.21***	0.02	0.21***	0.33***	0.44***	-						
	[10]Resting Habits	0.09**	0.23***	0.23***	0.21***	-0.03	0.21***	0.32***	0.38***	0.47***	-					
	[11]Tobacco use	-0.01	-0.16***	-0.04	-0.06	0.04	0.03	-0.06	-0.13***	-0.14***	-0.13***	-				
	[12]Alcohol consumption	-0.00	-0.21***	0.00	0.01	0.03	-0.01	-0.05	-0.18***	-0.20***	-0.21***	0.58***	-			
	[13]Use of other drugs	-0.02	-0.20***	-0.03	-0.03	0.06	-0.00	-0.07*	-0.15***	-0.19***	-0.21***	0.68***	0.63***	-		
	[14]Physical activity	0.05	0.09*	0.27***	0.22***	0.02	0.17***	0.20***	0.35***	0.28***	0.22***	-0.01	-0.02	-0.02	-	
Satisfaction with life (EVS)	[15]Satisfaction with life	0.10**	0.19***	0.39***	0.39***	-0.01	0.35***	0.47***	0.33***	0.37***	0.43***	-0.15***	-0.15***	-0.20***	0.23***	-
	Mean	55.98	48.91	32.78	20.57	26.76	24.34	25.12	9.32	9.90	9.46	4.40	8.28	7.72	17.19	16.33
	SD	9.62	6.62	4.66	4.52	7.50	7.10	7.16	2.65	3.16	3.16	2.81	4.32	4.15	5.10	4.49
	Min.	21	27	19	8	8	8	8	3	3	3	3	5	5	5	5
	Max.	75	77	44	32	40	40	40	15	15	15	15	24	25	25	25

***p < 0.001; **p < 0.01; *p < 0.05; PCQ = Prosocial Behaviours Questionnaire; TMMS-24 = *Trait Meta-Mood Scale* (Emotional Intelligence); HLS-II = Healthy Lifestyles Questionnaire; ESV = Satisfaction with Life Scale

About the results of emotional intelligence, it should be noted that this variable is related to all the dimensions of prosocial behaviour (empathy, respect, social relations and leadership) as mentioned above. As for the associations between emotional intelligence and healthy lifestyle, it is noteworthy that there is no relationship between the emotional attention factor and any of the dimensions of healthy lifestyle. On the other hand, the emotional clarity factor is related to healthy diet, respect for mealtimes, rest habits and physical activity. Finally, the emotional repair factor is positively related to healthy diet, respect for mealtimes, rest habits and physical activity and negatively related to the use of other drugs. A relationship is associated between life satisfaction and the factors of emotional clarity and emotional repair.

Finally, the relationships found between healthy lifestyle and life satisfaction were significantly positive in all dimensions of healthy lifestyle (healthy diet, respect for mealtimes, rest habits and physical activity) except for the consumption dimensions (tobacco use, alcohol use and use of other drugs) which were negative.

### Violence and consumption as risk factors

The data in Table 2 show how adolescents who do not bully their peers show higher levels of empathy and respect. Notably, those who are not victims have higher levels of social relationships. On the other hand, those who are observers of violence are more empathetic compared to non-observers who show greater respect.

**Table 2:** Differences between prosocial behaviours and roles of violence (girls *n* = 377; boys *n* = 366)

Roles of violence			Prosocial Behaviours			
			Empathy	Respect	Social Relations	Leadership
Aggressor	Aggressor	*Mean*	53.13	44.13	31.98	21.07
		*SD*	10.74	7.54	4.94	4.76
	Non-aggressor	*Mean*	56.22	49.33	32.84	20.53
		*SD*	9.41	6.39	4.65	4.52
	*t*		-2.34*	-5.80***	-1.32	0.86
	*p*		0.019	< 0.001	0.185	0.388
	*d*		0.17	0.43	-	-
Victim	Victim	*Mean*	57.12	47.91	31.63	20.48
		*SD*	10.04	7.21	4.95	4.90
	Non-victim	*Mean*	55.90	49.13	32.96	20.60
		*SD*	9.45	6.49	4.61	4.50
	*t*		1.17	-1.57	-2.62**	-0.25
	*p*		0.241	0.118	0.009	0.796
	*d*		-	-	0.19	-
Observer	Observer	*Mean*	57.04	48.27	32.74	20.86
		*SD*	9.62	6.81	4.45	4.60
	Non-observer	*Mean*	55.29	49.60	32.82	20.32
		*SD*	9.26	6.40	4.84	4.47
	*t*		2.50*	-2.70**	-0.23	1.59
	*p*		0.013	0.007	0.812	0.111
	*d*		0.18	0.20	-	-

***p < 0.001; **p < 0.01; *p < 0.05

Table 3 shows that adolescents who do not use tobacco or alcohol show more respect in their social interactions than those who do.

**Table 3:** Differences between prosocial behaviour and substance use (girls *n* = 377; boys *n* = 366)

Consumption			Prosocial Behaviours			
			Empathy	Respect	Social Relations	Leadership
Tobacco	Consumer	*Mean*	56.28	46.94	32.45	20.17
		*SD*	9.53	6.62	4.34	4.73
	Non-consumer	*Mean*	55.82	49.60	32.90	20.71
		*SD*	9.69	6.49	4.77	4.44
	*t*		0.56	-4.87***	-1.13	-1.42
	*p*		0.571	< 0.001	0.255	0.155
	*d*		-	0.36	-	-
Alcohol	Consumer	*Mean*	55.76	47.83	32.82	20.57
		*SD*	9.49	6.37	4.36	4.51
	Non-consumer	*Mean*	55.90	50.43	32.60	20.55
		*SD*	9.92	6.67	5.08	4.47
	*t*		-0.18	-5.21***	0.59	0.05
	*p*		0.853	< 0.001	0.552	0.958
	*d*		-	0.38	-	-

***p < 0.001

Table 4 provides data on the different dimensions of emotional intelligence (attention, clarity and repair) and the different roles of violence (bully, victim and observer). The results indicate that adolescents who do not engage in bullying situations have greater emotional repair than those who are bullies. On the other hand, observers of violence have a higher mean score in emotional attention, while non-observers score higher in emotional repair. No significant differences were found for victims of bullying.

**Table 4:** Differences between emotional intelligence and violence roles (girls *n* = 377; boys *n* = 366)

Roles of violence			Emotional intelligence		
			Attention	Clarity	Repair
Aggressor	Aggressor	*Mean*	25.90	23.23	22.31
		*SD*	8.65	8.28	7.49
	Non-aggressor	*Mean*	26.81	24.48	25.34
		*SD*	7.39	7.00	7.10
	*t*		-0.76	-1.28	-3.08**
	*p*		0.445	0.200	0.002
	*d*		-	-	0.23
Victim	Victim	*Mean*	27.59	23.90	24.57
		*SD*	7.39	8.00	7.81
	Non-victim	*Mean*	26.66	24.48	25.25
		*SD*	7.47	6.97	7.06
	*t*		1.14	-0.74	-0.86
	*p*		0.254	0.458	0.389
	*d*		-	-	-
Observer	bserver	*Mean*	27.48	24.21	24.47
		*SD*	7.39	7.20	7.23
	Non-observer	*Mean*	26.11	24.50	25.65
		*SD*	7.47	7.03	7.05
	*t*		2.47	-0.56	-2.22
	*p*		0.013*	0.576	0.026*
	*d*		0.18	-	0.16

**p < 0.01; *p < 0.05

Referring to the differences obtained between emotional intelligence and alcohol and tobacco consumption (Table 5), it is worth noting that adolescents who do not consume either of these two substances have greater emotional repair than those who do consume.

**Table 5:** Differences between emotional intelligence and substance use (girls *n* = 377; boys *n* = 366)

Consumption			Emotional intelligence		
			Attention	Clarity	Repair
Tobacco	Consumer	*Mean*	27.69	23.97	23.97
		*SD*	7.63	7.32	7.11
	Non-consumer	*Mean*	26.45	24.52	25.61
		*SD*	7.47	7.04	7.10
	*t*		1.95	-0.92	-2.75**
	*p*		0.051	0.353	0.006
	*d*		-	-	0.20
Alcohol	Consumer	*Mean*	26.93	24.12	24.64
		*SD*	7.53	7.18	7.00
	Non-consumer	*Mean*	26.45	24.77	26.11
		*SD*	7.51	6.86	7.13
	*t*		0.82	-1.19	-2.71**
	*p*		0.408	0.232	0.007
	*d*		-	-	0.20

**p < 0.01

### Healthy habits and substance use: profiles and differences in prosocial behaviour and emotional intelligence

**Fig. 1:**
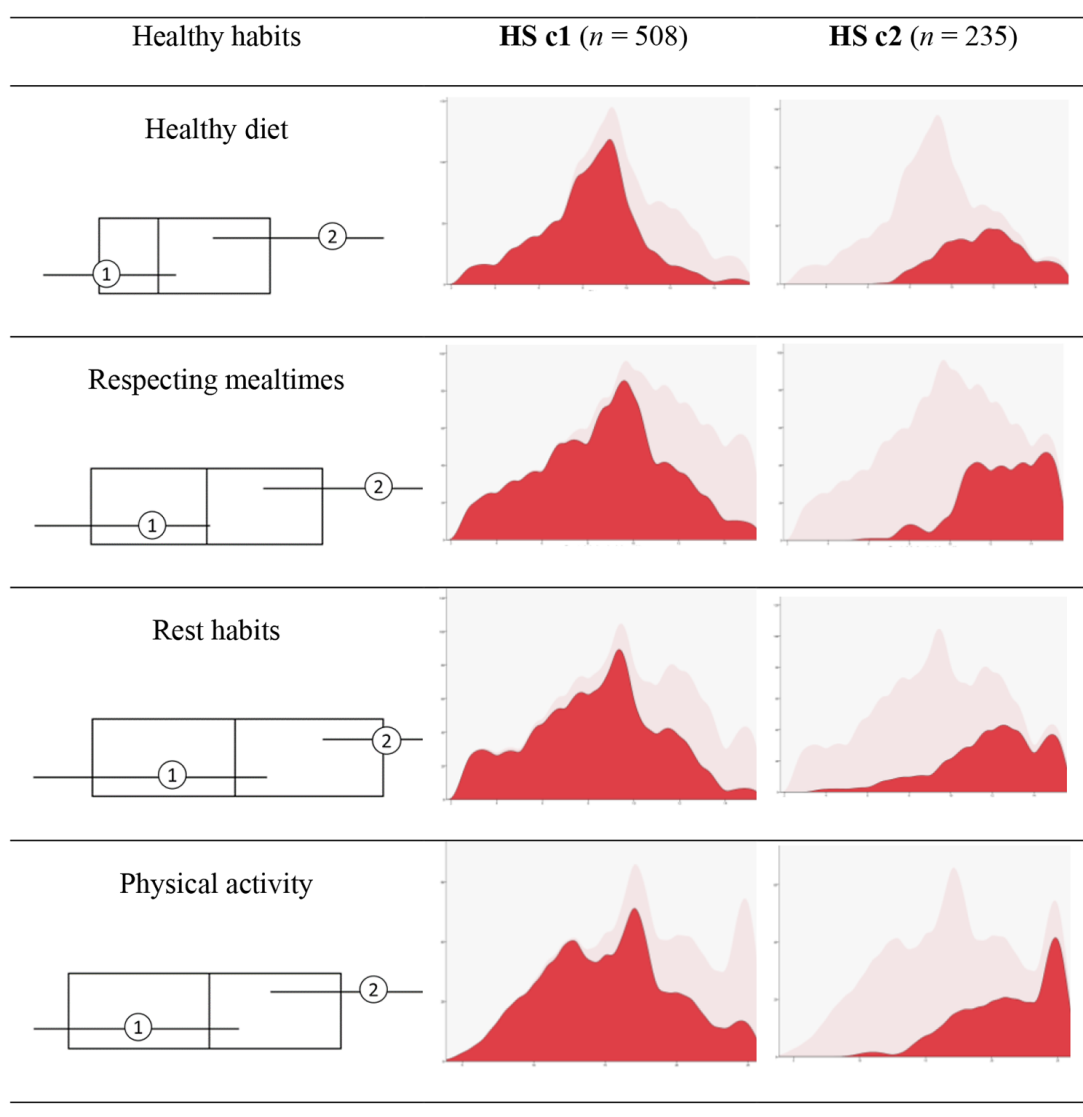
Composition of healthy behaviour clusters

First, to identify profiles based on healthy habits, a two-stage cluster analysis is carried out with four entries (healthy diet, respecting mealtimes, rest habits, and physical activity), from which two groups or clusters are obtained (Fig. 1). In this case, as a measure of cluster quality, an average silhouette of 0.4 is obtained.

The first cluster (HSc1), consisting of 68.4% of the cases (*n* = 508), is characterised by scores below the overall mean in the dimensions: healthy diet (*M* = 8.28), respecting mealtimes (*M* = 8.67), rest habits (*M* = 8.33), and physical activity (*M* = 15.32).

The second cluster (HSc2), with 31.6% of cases (*n* = 235), is defined by above sample mean scores on healthy diet (*M* = 11.56), respecting mealtimes (*M* = 12.58), resting habits (*M* = 11.90), and physical activity (*M* = 21.23).

Table 6 presents the descriptive data for both clusters and the results of the comparison of means between the profiles in prosocial behaviour and emotional intelligence. These results indicate that adolescents with high scores on healthy diet, respect for mealtimes, rest habits and physical activity (HSc2) have greater empathy, respect, social relationships, leadership, clarity and emotional repair.

**Table 6:** Prosocial behaviour and emotional intelligence. Descriptive data and t-test according to healthy habits profile

			Healthy habits						*t*	*p*	*d*
			HSc1			HSc2					
			*N*	*M*	*SD*	*N*	*M*	*SD*			
Prosocial Behaviour	Empathy		508	55.48	9.94	235	57.05	8,79	-2.16*	0.031	0.17
	Respect		508	47.96	6.59	235	50.96	6,23	-5.87***	< 0.001	0.46
	Social relations		508	31.93	4.67	235	34.61	4,07	-7.96***	< 0.001	0.63
	Leadership		508	19.99	4.42	235	21.82	4,51	-5.20***	< 0.001	0.41
Emotional Intelligence	Emotional Attention		508	26.81	7.82	235	26.67	6,77	0.24	0.804	-
	Emotional Clarity		508	23.37	7.08	235	26.42	6,68	-5.55***	< 0.001	0.44
	Emotional Repair		508	23.79	7.08	235	27.97	6,46	-7.68***	< 0.001	0.61

***p < 0.001; *p < 0.05

**Fig. 2:**
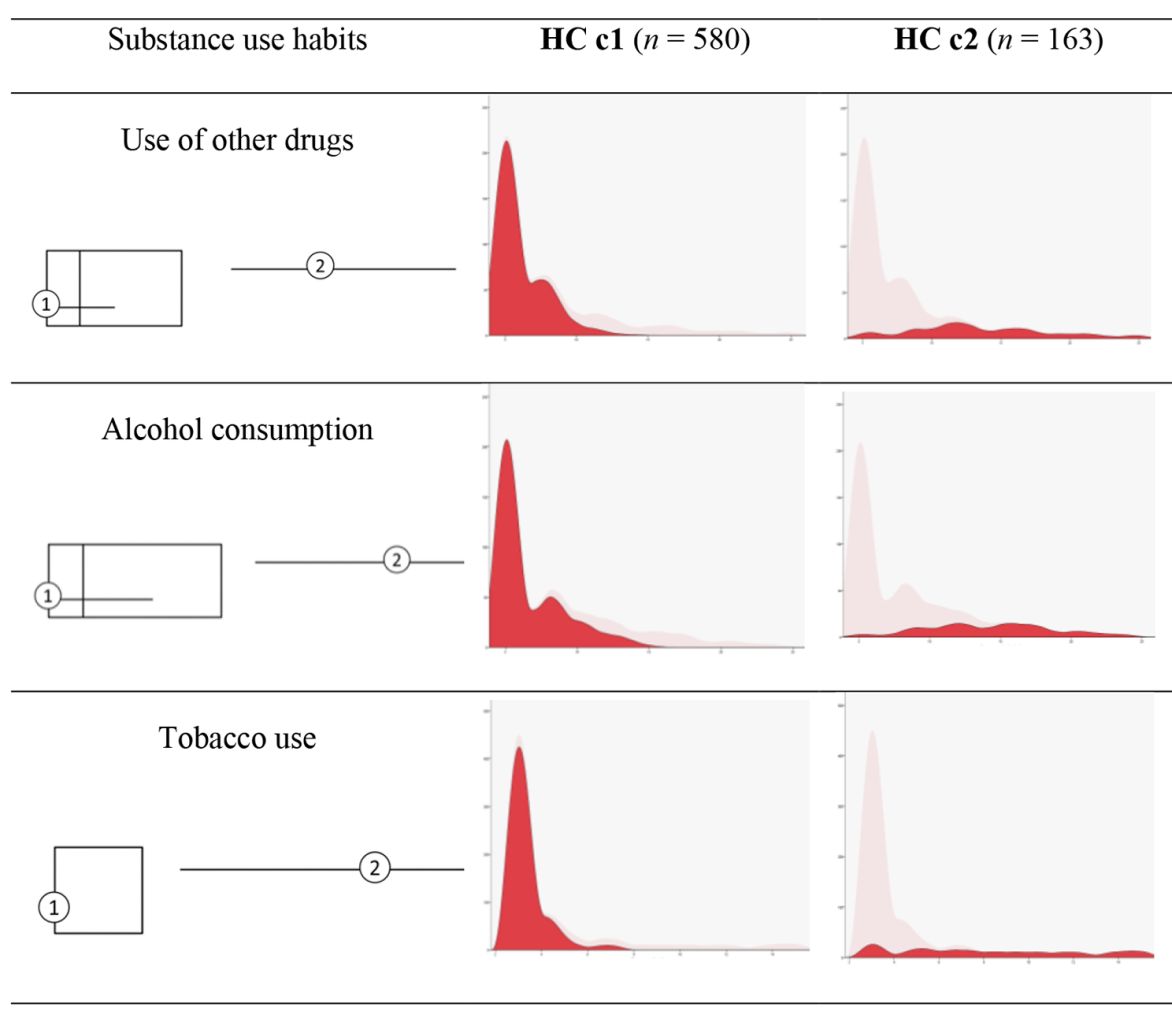
Composition of substance use clusters

On the other hand, to identify profiles based on substance use habits, a two-stage cluster analysis is carried out with three entries referring to alcohol, tobacco and other drug use. By automatic grouping, two clusters are obtained (Fig. 2). In this case, as a measure of cluster quality, an average silhouette of 0.7 is obtained.

The first cluster (HCc1), consisting of 78.1% of the cases (*n* = 580), is characterised by scores below the overall mean for alcohol (*M* = 6.58), tobacco (*M* = 3.33), and other drugs (*M* = 6.06).

The second cluster (HCc2), with 21.9% of cases (*n* = 163), is defined by above sample mean scores on alcohol (*M* = 14.30), tobacco (*M* = 8.21), and other drugs (*M* = 13.60).

Table 7 presents the descriptive data for both clusters and the results of the comparison of means between the profiles in prosocial behaviour and emotional intelligence. These results show that adolescents with a lower consumption of alcohol, tobacco and other drugs (HCc1) score higher on respect.

**Table 7:** Prosocial behaviour and emotional intelligence. Descriptive data and t-test according to substance use habits profile

			Substance use habits						*t*	*p*	*d*
			HCc1			HCc2					
			*N*	*M*	*SD*	*N*	*M*	*SD*			
Prosocial Behaviour	Empathy		580	55.99	9.63	163	55.92	9.58	0.08	0.933	-
	Respect		580	49.47	6.48	163	46.90	6.77	4.41***	< 0.001	0.68
	Social relations		580	32.88	4.64	163	32.41	4.71	1.13	0.256	-
	Leadership		580	20.68	4.42	163	20.17	4.86	1.25	0.211	-
Emotional Intelligence	Emotional Attention		580	26.51	7.45	163	27.65	7.64	-1.71	0.087	-
	Emotional Clarity		580	24.24	7.01	163	24.68	7.41	-0.68	0.492	-
	Emotional Repair		580	25.31	7.07	163	24.42	7.45	1.40	0.162	-

***p < 0.001

### Probability of a healthy lifestyle and substance use by age, gender, prosocial behaviour and emotional intelligence

A bilateral logistic regression analysis was carried out to determine the probability that adolescents lead a healthy lifestyle and that they consume substances such as alcohol, tobacco or other drugs according to different variables such as age, sex, prosocial behaviour and emotional intelligence.

Table 8 shows how gender, namely being male, respect, social relationships and emotional repair are more likely to lead a healthy lifestyle.

**Table 8:** Likelihood of having a healthy life according to the variables examined (Total sample; *N* = 743)

Healthy lifestyle habits						
**Variables**	**ß**	**Standard error**	**Sig.**	**Exp (ß)**	**95% C.I. for EXP (ß)**	
					**Inferior**	**Superior**
Sex (Male)	0.80	0.18	<0.001	2.22	1.54	3.21
Age	-0.35	0.10	<0.001	0.70	0.56	0.86
Empathy (CP)	-0.02	0.01	0.038	0.97	0.94	0.99
Respect (CP)	0.07	0.01	<0.001	1.072	1.03	1.10
Social relations (PC)	0.08	0.02	<0.001	1.092	1.04	1.14
Leadership (CP)	0.02	0.02	0.425	1.020	0.97	1.07
Emotional Attention (I.E)	-0.01	0.01	0.380	0.98	0.96	1.01
Emotional Clarity (E.C)	0.00	0.01	0.644	1.00	0.97	1.03
Emotional Repair (E.R.)	0.05	0.01	0.001	1.05	1.02	1.08
Constant	-2.35	1.77	0.186	0.09		

The probability of having a substance abuse habit (Table 9) is linked to the age of the adolescents, as well as to the empathy dimension in prosocial behaviour and emotional clarity in emotional intelligence.

**Table 9:** Likelihood of substance use according to the variables tested (Total sample; *N =* 743)

Substance use habits						
**Variables**	**ß**	**Standard error**	**Sig.**	**Exp (ß)**	**95% C.I. for EXP (ß)**	
					**Inferior**	**Superior**
Sex (Male)	-0.25	0.19	0.206	0.77	0.52	1.14
Age	0.44	0.10	<0.001	1.55	1.26	1.91
Empathy (CP)	0.03	0.01	0.008	1.03	1.00	1.06
Respect (CP)	-0.09	0.01	<0.001	0.90	0.87	0.93
Social relations (PC)	-0.00	0.02	0.971	0.99	0.95	1.05
Leadership (CP)	-0.05	0.02	0.060	0.95	0.90	1.00
Emotional Attention (I.E)	0.01	0.01	0.256	1.01	0.98	1.04
Emotional Clarity (E.C)	0.03	0.01	0.025	1.03	1.00	1.07
Emotional Repair (E.R.)	-0.01	0.01	0.559	0.99	0.95	1.02
Constant	-5.11	1.79	0.004	0.00		

## Discussion and conclusions

This research has revealed the relationships between prosocial behaviour and emotional intelligence with a healthy lifestyle and life satisfaction in adolescence, as well as their influence on school violence and the consumption of substances such as tobacco and alcohol.

Hypothesis 1 of this study that prosocial behaviours and emotional intelligence are positively associated with healthy lifestyle habits and life satisfaction has been accepted. Previous scientific literature points out how prosocial behaviours are a positive element in adolescence, due to the fact that it helps socialisation, a more positive mood and social and personal well-being [7, 8]. This statement corresponds to the results obtained in our research, because a positive correlation was found between the different dimensions of prosocial behaviours and life satisfaction. In terms of healthy lifestyle, it was found that respect, social relations and leadership are related to physical activity, since, as Li & Shao [9] state, sport activities influence the promotion of prosocial values, among others. This positive association has also been found in other healthy lifestyle factors such as healthy diet, respect for mealtimes and rest habits, because healthy eating is a factor that is influenced by prosocial behaviours [10]. Regarding emotional intelligence, the results obtained indicate a significant relationship of this construct with all dimensions of prosocial behaviour and with life satisfaction. These data are in line with studies indicating that adolescents with a good level of emotional intelligence are more satisfied with life and have greater self-esteem and happiness [16–18]. We have also found that certain dimensions of emotional intelligence such as repair and clarity correlate positively with several elements of a healthy lifestyle (healthy diet, respect for mealtimes, rest habits and physical activity), suggesting that emotional intelligence promotes a healthy lifestyle and vice versa.

Hypothesis 2 of this research on the existence of differences in school violence and substance use according to prosocial behaviour and emotional intelligence has also been accepted. The results obtained on prosocial behaviour and peer violence indicate that non-bullies have greater respect and empathy. This idea is supported by other studies, which indicate that prosocial behaviour mediates violence and that bullies do not tend to engage in prosocial behaviour [12, 13]. In addition, observers of violence show greater empathy, while non-observers have greater respect; on the contrary, adolescents who are not victims tend to have greater social relationships. All these ideas may be associated with the fact that, as stated in the study by Manzano-Sánchez et al. [14], prosocial behaviour is negatively related to violence. In terms of consumption, the results show a negative relationship between respect and the consumption of alcohol, tobacco and other drugs, with those who consume the least being those who have the highest scores in respect; since, as other studies have shown, prosocial adolescents have a lower consumption of these substances [11]. On the other hand, studies such as that of León del Barco et al. [20] indicate that emotional intelligence acts as a protective factor against school violence, and we can corroborate this assertion since our results show that adolescents who do not bully and who have not observed violent situations among their peers have greater emotional repair. Such ideas are also shown in other studies that indicate that emotional intelligence is negatively related to violence [21, 22]. As with substance use, because non-users of neither tobacco nor alcohol also have a higher level of emotional repair, so, as other studies indicate, emotional intelligence may act as a mediator with consumption in adolescence [23].

For hypothesis 3, a two-stage cluster analysis was carried out to obtain two groups for healthy lifestyle habits (HSc1 = unhealthy group; HSc2 = healthy group) and another two for substance use (HCc1 = non-users; HCc2 = users). The results obtained confirm the initial hypothesis, since differences have been obtained between these groups with respect to prosocial behaviours and emotional intelligence. The healthy living group (HSc2) scored higher on empathy, respect, social relationships, leadership, clarity and emotional repair compared to the unhealthy group (HSc1). These results are linked to the fact that personal well-being promotes positive behaviours and self-esteem [5]. Prosocial behaviours and emotional intelligence are positively associated with aspects related to healthy living, physical activity and social and personal well-being [7, 10, 19]. On the other hand, the group of non-consumers (HCc1) obtained higher levels of respect than consumers (HCc2), which may be due to the fact that emotional intelligence mediates consumption [23].

Hypothesis 4 aimed to find out which dimensions of prosocial behaviour and emotional intelligence are most related to having a healthy life or having substance consumption habits. To this end, a bilateral logistic regression analysis was carried out which indicates that being male, having respect, social relationships and emotional repair encourage the possibility of having a healthy life. This may be linked to the fact that a high level of emotional intelligence and prosocial behaviours provides the right tools to lead a healthy life and promote personal well-being [5, 6]. On the other hand, it has been found that age, empathy and emotional clarity are the factors that may be most at risk when it comes to consuming harmful substances. This assessment may be because in adolescence, consumption tends to take place in a context of leisure and socialisation. In addition, aspects such as empathy and stress are considered to be factors that may lead to substance use [4].

In conclusion, this research details how the promotion of pro-social behaviours and emotional intelligence in adolescence helps young people to lead healthier lives, reduces peer violence and the consumption of harmful substances and, therefore, favours life satisfaction and personal well-being. The practical implications of this study are to know which variables are positive in adolescence and need to be promoted in our young people in order to reduce the risk factors inherent to this stage of change and vulnerability. In terms of limitations, it is important to highlight that although this study addressed the roles of peer violence, it was dealt with in a general way. It should be borne in mind that often the same student may play several roles at the same time, which may influence the interpretation of the results. Furthermore, another limitation is related to how students’ consumption has been assessed. It is considered important to propose other data on the context of consumption that would be relevant, given that only whether or not participants consume and not the context in which this consumption takes place has been analysed. This limitation deprived the study of relevant information on the circumstances and motivations underlying these behaviours. It is considered important to propose other data on the context of consumption that would be relevant, given that we only analysed whether or not participants consume and not the context in which this consumption occurs. In relation to these limitations and as future lines of research, it would be interesting to delve deeper into the roles of peer violence, taking into account the multiplicity of roles that an individual can assume. Furthermore, it would be enlightening to analyse specifically the type of violence exercised in these roles in order to better understand their dynamics and consequences. On the other hand, it would also be beneficial to broaden the analysis of substance use, incorporating details about the context, motivations and environmental factors surrounding this behaviour, which could provide a more complete and accurate understanding. Thus, it is proposed to explore the interaction between the promotion of prosocial behaviours and emotional intelligence in adolescence with other relevant factors, such as the family, school and community environment. Such future research could foster the design of more personalised programmes that comprehensively address the developmental challenges faced by young people. We conclude by highlighting the effectiveness of promoting prosocial behaviour and emotional intelligence in adolescence and its positive effects on certain current problems such as school violence and substance abuse.

## Data Availability

Data without participant identification in this study can be obtained by emailing the corresponding author.
